# The Dynamics of the Interrelationships between Conscious Self-regulation, Psychological Well-being and School-related Subjective Well-being in Adolescents: A Three-year Cross-lagged Panel Study

**DOI:** 10.11621/pir.2021.0303

**Published:** 2021-09-15

**Authors:** Varvara I. Morosanova, Tatiana G. Fomina, Irina N. Bondarenko

**Affiliations:** a Psychological Institute of the Russian Academy of Education, Moscow, Russia

**Keywords:** conscious self-regulation, subjective well-being, psychological well-being, cross-lagged analysis, adolescents.

## Abstract

**Background:**

Recently, research on psychological well-being and its dynamics and predictors in adolescence, has gained special attention, due to the importance of well-being for mental and physical health, as well as for success in different activities. Self-regulation (SR) is considered a significant resource for maintaining psychological and school-related subjective well-being.

**Objective:**

The purpose of our study was to identify the role of conscious SR in maintaining pupils’ satisfaction with school life, and to assess the contribution of conscious SR to the development of psychological well-being in adolescence.

**Design:**

Two three-year longitudinal studies were carried out on samples of young adolescents in Russian schools (N = 148; N = 132; 10–13 years). The studies utilized methods for assessing conscious SR, psychological well-being (PWB), and school-related subjective well-being (SWB), the latter being the cognitive component of life satisfaction.

**Results:**

Our research revealed differences in the dynamics of PWB and SWB levels in adolescents during their transition from primary to basic secondary school. It also identified the specifics of longitudinal relationships between conscious SR, PWB, and SWB in adolescence. We showed that there was a reciprocal relationship between them. However, the most significant cross-longitudinal effects were established between SR and school-related SWB. These effects changed over time: at the beginning, well-being acted as a significant factor of self-regulation, while later self-regulation acted as a significant resource for maintaining adolescent well-being in the subsequent years.

**Conclusion:**

School-related SWB is characterized by the most pronounced trajectory of change, while PWB is characterized by greater stability and insignificant growth. Our three-year longitudinal study demonstrated that the link between self-regulation and well-being is consistently reproduced. Conscious self-regulation is a significant resource for both the psychological and school subjective well-being of adolescents.

## Introduction

Research on psychological well-being (PWB) is a significant trend in today’s studies of education (Eryilmaz, 2012; Ronen, Hamama, Rosenbaum, & Mishely-Yarlap, 2016; Steinmayr, Wirthwein, Modler, & Barry, 2019). It has been shown that a high level of psychological well-being has a positive effect on academic performance, school engagement, academic self-efficacy, and social adaptation, in addition to reducing the level of academic stress (Antaramian, 2017; Suldo, Gormley, Dupaul, & Anderson-Butcher., 2014; Thomaes, Sedikides, van den Bos, Hutteman, & Reijntjes, 2017). Longitudinal studies of PWB-related factors are of particular importance (Converse, Beverage, Vaghef, & Moore, 2018; Steinmayr, Heyder, Naumburg, Michels, & Wirth-wein, 2018; Yang, Tian, Huebner, & Zhu, 2019).

However, predictors of PWB itself have been studied to a lesser extent. There is ample evidence that conscious self-regulation of achieving educational goals is a significant factor in both academic success and PWB (Gestsdottir & Lerner, 2008; Fomina, Burmistrova-Savenkova, & Morosanova, 2020; Singh, & Sharma, 2018). The purpose of this article is to uncover the dynamics of conscious self-regulation as a significant resource for schoolchildren’s well-being.

### Psychological Well-being and Subjective well-being

In modern research, the well-being of schoolchildren is assessed by means of various measures. The most theoretically and empirically substantiated are the constructs of psychological well-being and subjective well-being. These are interrelated but somewhat different indicators of positive human functioning (Ryan & Deci, 2001). SWB includes a cognitive component, which is reflected as a self-assessment of overall satisfaction with one’s life, and an affective component, which is measured as a balance between positive and negative emotions (Diener, 1999). The cognitive component is considered as the most stable component in the SWB structure and is analyzed more often.

It’s worth emphasizing that SWB is usually considered in connection with certain specific areas of life. Thus, it seemed relevant for us to focus on the study of school-related subjective well-being, which is characterized by students’ satisfaction with their success at school, their relationships with teachers and peers, the school climate, etc. (*e.g.,* Tian, Tian, & Huebner, 2016; Steinmayr et al., 2018; Yang et al., 2019).

On the other hand, psychological well-being (PWB) is based on a person’s holistic experience, such as feelings of happiness, and satisfaction with oneself and one’s life in the broader context of relations between oneself and the world (Huppert, 2009; Ryff, 1989; Waterman, 1993, etc.). PWB is a multidimensional phenomenon. Its elements are: 1) self-acceptance; 2) positive relations with others; 3) autonomy; 4) environmental mastery; 5) purpose in life, and, finally, 6) personal growth. These six constructs define PWB both theoretically and operationally, and they specify what promotes emotional and physical health (Ryff& Singer 1998).

Although there have been recent attempts to discover a common factor determining SWB and PWB (Garcia, Sagone, De Caroli, & Al Nima, 2017; Heintzelman, 2018), the most promising results have been obtained by differentiating between them (Ryan and Deci, 2001). Indeed, as a rule, factor analysis confirms a close but still different status of SWB and PWB (Compton, Smith, Cornish, & Qualls, 1996; Keyes, Shmotkin, & Ryff, 2002).

The current scientific consensus on the relationship between PWB and SWB research can be described as follows: “PWB and SWB are strongly related at the general construct level, but their individual components are distinct once their overlap with the general construct of well-being is partially led out.” (Chen, Jing, Hayes, & Lee, 2013). We adhered to this position in this study. Our main concern was to compare the specific relationships of conscious self-regulation with both PWB and the cognitive component of SWB in adolescents during their transition from primary to basic secondary school.

Self-regulation (SR), as considered in different contexts by empirical studies, is associated with both PWB and SWB. (*e.g*, Elliot, Thrash, & Murayama, 2011; Fomina et al., 2020; Hofer, Busch, & Kartner, 2011; Wrosch, Scheier, Miller, Schulz, & Carver, 2003; Tavakolizadeh, 2012). It has been shown that the higher a person’s SR, the higher their sense of well-being, and the more effective their coping strategies (Boekaerts & Corno, 2005; Hofer et al., 2011; Saha, Huebner, Hills, Malone, & Valois, 2014). A study by Saha and colleagues (2014) demonstrated that SR explains a significant percentage of the variance across all six PWB measures, with the greatest positive associations found between SR and life goals. It’s worth noting that all these data were obtained on samples of university students or adults. There are very few similar studies on the relationships of SR with PWB and SWB conducted on the samples of adolescents (Steinmayr et al., 2019). Longitudinal studies can make a significant contribution to uncovering the relationships between conscious SR, PWB, and SWB, since there are data on different trajectories of PWB and SWB in adolescents (Archakova, Veraksa, Zotova, & Perelygina, 2017).

In our approach, the conscious self-regulation of learning activity is understood as a cognitive-personal construct, including cognitive processes (planning goals, modeling significant conditions for goals achievement, programming actions, and evaluating results) and regulatory-intrapersonal properties (flexibility, independence, reliability, and responsibility), which serve as tools for initiating and maintaining activity aimed at consciously setting educational goals and managing their achievement (Morosanova, 2004-2020). Conscious SR is the controlling mechanism for mobilizing all other types of individual resources (cognitive, motivational, and intrapersonal) to achieve a result (Morosanova, 2014). Success, reliability, productivity, and the final result of actions to achieve the goal depend on the level of development of operational-regulatory processes and regulatory-personal features.

Adolescence has traditionally been associated with the risk of behavioral problems and psychological distress. In this connection we considered it extremely important to study the contribution of SR to PWB and SWB, particularly in early adolescence, when children’s sense of well-being is of maximum importance; it gradually decreases later toward high school. Empirical studies show that the period between ages 10 and 12 years is a turning point in the development of individual trajectories of PWB and SWB (Orben, Lucas, Fuhrmann, & Kievit, 2020; Willroth, Atherton, & Robins, 2020). At the same time, according to a number of researchers, personal changes during adolescence create unique opportunities for positive trajectories of development (Lerner et al., 2018). Herewith, SR serves as one of the essential mechanisms contributing to positive youth development (Gestsdottir et al., 2017).

The reflexivity that takes shape during this period probably facilitates the ability of adolescents to develop such subjective qualities as independence, responsibility, and initiative, which make their significant contributions to adolescent well-being. We assumed that conscious self-regulation, being the control level of regulation of educational goals achievement, would determine the level and dynamics of PWB and SWB during this period.

Our research was aimed to answer the following questions:

1. What are the dynamics and specificity of the relationship between conscious self-regulation, psychological well-being, and school-related subjective wellbeing of adolescents during their transition from primary to basic secondary school?
2. Can conscious SR be considered a long-term predictor of PWB and SWB in adolescents during this transition period?

For research purposes, we conducted two studies. The data obtained and results of the analysis are presented and discussed below.

## Methods

### Participants and Procedure

Two separate longitudinal studies were conducted on samples of teenagers (grades 4–6) in Russian state schools which implement the basic education program. The 4th grade in Russia is the last year of primary school. Then children go to the basic secondary school. The design of the studies differed in the instruments for assessing the schoolchildren’s well-being: in the first study, the methodology for assessing psychological well-being (PWB) was used; in the second, we used the scale for assessing the school-related subjective well-being (SWB).

The data were collected in three waves. In Study 1 the sample at T1, T2, and T3 consisted of 148 students. The sample was evenly distributed by sex (50% boys). At T1, the mean age of the participants was 10.2 years (SD = 0.50; range = 10–11 years). Seven months later, at T2 children were on average 10.9 years old (SD = 0.28; range = 10–12 years). One year later, at T3 children were on average 11.9 years (SD = 0.50; range = 12–13 years). In Study 2 the sample at T1, T2, and T3 consisted of 132 pupils (47% boys). At T1, the mean age of the participants was 10.3 years (SD = 0.48; range = 10–11 years). Seven month later, at T2 children were on average 10.8 years old (SD = 0.42; range = 10–12 years). One year later, at T3 children were on average 11.9 years old (SD = 0.32; range = 12–13 years).

Parental and school consent was obtained for all participants. Analyses were carried out on depersonalized data. The study procedure was approved by the relevant institutional review board. Ethical agreement and consent for access to the schools were provided by the Ethics Committee of the Psychological Institute of the Russian Academy of Education (approval number 2017/1-128).

### Measures

***Psychological Well-Being*** was accessed using the slightly modified Russian adaptation of the 25-item Well-Being Manifestation Measure Scale developed by Masse (Masse, Poulin, Dassa, Lambert, Bélair, & Battaglini, 1998). The questionnaire was previously validated on a sample of 4th-grade pupils in Russian secondary schools (Morosanova, Bondarenko & Fomina, 2018). The participants were asked to evaluate to what extent they experienced the described states over the past month on a 5-point Likert scale ranging from 1 (never) to 5 (almost always).

This questionnaire contained the following subscales: 1) Control of Self and Events (*e.g.*, “I was able to face difficult situations in a positive way”); 2) Happiness (*e.g.*, “I found life exciting and I wanted to enjoy every moment of it”); 3) Social Involvement (*e.g.,* “I felt like having fun, doing sports and participating in all my favorite activities and pass-times”); 4) Self-Esteem (*e.g.*, “I had self-confidence”); 5) Mental Balance (*e.g.*, “My life was well-balanced between my family, personal and school activities”); 6) Sociability (*e.g.*, “I got along well with everyone around me”); and the cumulative scale 7) Psychological Well-Being, which summed up the scores on all the scales. The internal reliability coefficients were 0.72–0.78.

***School-related Subjective Well-Being*** was measured by means of the Multidimensional Students Life Satisfaction Scale (MSLSS) (Huebner, 2001, in a Russian adaptation by Sychev, Gordeeva, Lunkina, Osin, & Sidneva, 2018). Its 30 items allow for evaluating schoolchildren’s satisfaction in important life domains, including family, school, self, friends, and teachers, on a 5-point Likert scale ranging from 1 (never) to 5 (always). Higher scores indicate higher levels of life satisfaction throughout the scale. All the scales had high reliability (0.82 < α < 0.89).

***Self-Regulation*** was measured by means of Morosanova’s Self-Regulation Profile Questionnaire – Junior (Morosanova & Bondarenko, 2015). It consists of seven self-assessment scales: Planning of goals (*e.g.*, “I know what grades I want to get at the end of the year”); Modeling of significant conditions (*e.g.,* “Prior to start solving the task, I always carefully examine its introductory conditions”); Programming of Activity (*e.g.*, “I have no difficulty in drawing up a plan of presentation”); Results Evaluation (*e.g.,* “I rarely notice my mistakes”); Flexibility (*e.g.,* “I’m back to studies quickly after the holidays”); Independence (*e.g.*, “I usually do my homework by myself ”); and Responsibility (*e.g.,* “I seek to perform additional tasks”).

Each item was scored on a 6-point scale with responses ranging from 1 (“not at all like me”) to 6 (“very much like me”). The pupils were to choose to what extent the described behavior was characteristic of them. The general SR level was estimated by adding up the scores on the seven scales. The incentive material was presented in forms accessible for primary school age, such as descriptions of typical situations associated with organization of learning activities and pupils’ behavior relative to their training. The coefficients of internal consistency of the items for each scale ranged from 0.62 to 0.79, indicating an overall reasonable homogeneity of the items on each scale.

### Statistical Analysis

SPSS 26.0 (SPSS Inc.) was used to obtain descriptive statistics for the study variables and bivariate associations. Longitudinal confirmatory factor analyses and bidirectional cross-lagged panel analyses were conducted in AMOS 23. Two separate cross-lagged panel models were assessed, one between SR and PWB and the other between SR and school SWB. The models’ fits were evaluated using several fit indices: a Root Mean Square Error of Approximation (RMSEA); a comparative Fit Index (CFI); and the Tucker–Lewis index (TLI).

## Results

### Study 1. Dynamics of the relationship between psychological well-being and conscious self-regulation in adolescents: a cross-lagged panel analysis

**Figure 1.**
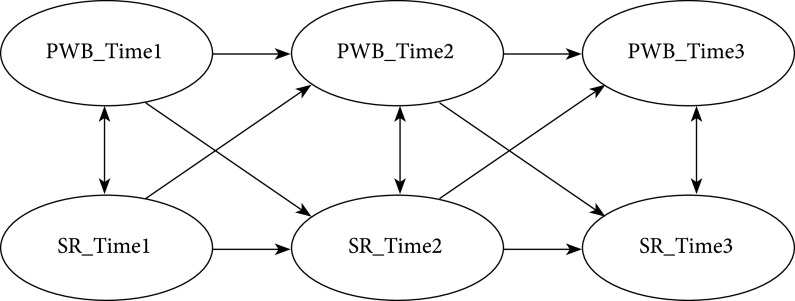
Cross-lagged models

*Note. The factor indicators are not shown for brevity*

The first study tested the hypotheses about the specificity of the longitudinal relationship between the conscious self-regulation and psychological well-being of adolescents. *Figure 1* shows the initial hypothesized model of this relationship.

The six PWB variables at each point in time were used as indicators of the latent factor PWB, labeled as PWB_Time1, PWB_Time2, and PWB_Time3. The latent factor SR (labeled as SR_Time1, SR_Time2, and SR_Time3) was represented by seven regulatory indicators.

The means, standard deviations, and correlations for Time 1, Time 2, Time 3 PWB, and SR are presented in *Table 1.* Analysis of significant changes in the levels of PWB and SR revealed that the PWB level significantly increased in the adolescents when they moved from the 4^th^ grade (Time 1) to the 5^th^ grade (Time 2), *i.e.*, from primary to basic secondary school. The same thing happened with SR (p = 0.023). But during their transition from grade 5 (Time 2) to grade 6 (Time 3), no significant changes in the levels of PWB and SR were recorded. Correlation analysis data indicated the presence of a moderate to strong relationship between the general level of psychological well-being and the general level of conscious self-regulation.

**Table 1:** Means, Standard Deviations, and Correlations among Variables (N=147)

	Mean	SD	1	2	3	4	5	6
1. PWB Time 1	93.71	15.247	1	.675**	.399**	.512**	.589**	.365**
2. PWB Time 2	95.90	14.125		1	.564**	.384**	.568**	.500**
3. PWB Time 3	96.47	15.126			1	.261**	.378**	.444**
4. SR Time 1	30.71	5.716				1	.595**	.498**
5. SR Time 2	31.29	5.007					1	.566**
6. SR Time 3	30.31	8.262						1

**p < .01, *p < .05. *Note. PWB = psychological well-being; SR = self-regulation*

At the next stage, to reveal the cross-longitudinal effects of PWB and SR, cross-lagged panel analyses were carried out using the method of structural modeling. The full model with all cross-lagged paths, auto-regressive paths, and concurrent covariance demonstrated an acceptable fit to the data (CFI= .983, TLI = .956, RM-SEA = .038). The path from PWB at Time 1 to SR at Time 2 was statistically significant. The paths from SR at Time 1 and Time 2 to PWB at Time 2 and Time 3 were all significant.

Next, we looked at the individual cross-longitudinal models. The structural models demonstrated a good level of agreement (see *Table 2*). This allows us to say that there was a significant reciprocal relationship between SR and PWB.

**Table 2:** Cross-lagged models

Model	X^2^	df	CFI	RMSEA	TLI
Cross-lagged (T1_SR → T2_PWB)	355.190	282	0.951	0.042	0.944
Cross-lagged (T1_PWB → T2_SR)	338.173	282	0.963	0.037	0.957
Cross-lagged (T2_PWB → T3_SR)	296.381	268	0.981	0.027	0.976
Cross-lagged (T2_SR → T3_PWB)	263.898	263	0.999	0.005	0.999

Then we compared the magnitude and significance of the standardized PWB and SR coefficients in the obtained models. Results for the auto- and cross-lagged analyses are presented in *Table 3.*

**Table 3:** Auto- and cross-lagged standardized path coefficients from the cross-lagged panel analyses

	PWB Time 2	PWB Time 3	SR Time 2	SR Time 3
PWB Time 1	0.70***		0.40***	
PWB Time 2	1	0.60***		***0.12***
SR Time 1	0.20*		0.53***	
SR Time 2		0.29**	1	0.83***

Notes. *p < 05.; **p < .01; ***p < .001. Cross-longitudinal coefficients are in bold italics

The results suggest that standardized coefficient of the self-regulation score is higher in the cross-longitudinal relationship model of SR (Time 2) and PWB (Time 3), while the standardized regression coefficients for PWB (Time 1) are higher in relation to SR (Time 2). These results indicated that PWB served as a firm foundation for the SR development at the stage of adolescent adaptation to secondary school (during the transition from primary school), and that after that, conscious SR (apparently having been formed in the new conditions) acts as a significant resource for maintaining PWB in adolescents in later years.

### Study 2. Dynamics of the relationship between school-related subjective well-being and conscious self-regulation in adolescents: a cross-lagged panel analysis

In the second study, we examined the specificity of the relationships between subjective school well-being and conscious self-regulation.

The means, standard deviations (SD), and correlations among the variables included in the analyses are presented in *Table 4.*

**Table 4:** Means, Standard Deviations, and Correlations among Variables (N= 132)

	Mean	SD	1	2	3	4	5	6
1. SWB Time 1	27.45	6.724	1	.561**	.420**	.447**	.223*	.333**
2. SWB Time 2	28.37	5.926		1	.480**	.217*	.424**	.273**
3. SWB Time 3	24.89	5.409			1	.277**	.377**	.392**
4. SR Time 1	29.89	5.770				1	.338**	.458**
5. SR Time 2	30.31	5.355					1	.588**
6. SR Time 3	30.23	5.317						1

**p <.01, *p <.05 *Note. SWB =subjective well-being; SR = self-regulation*

In contrast to PWB, we saw a somewhat different picture of the dynamics in relation to the school-related SWB. Younger adolescents, when moving from grade 4 (Time 1) to grade 5 (Time 2), demonstrated a small but significant positive trend (p = 0.048), and then a significant drop in their SWB level (p = 0.000). The dynamics of self-regulation corresponded to the results of Study 1.

The full model with all the cross-lagged paths, auto-regressive paths, and concurrent covariance also demonstrated an acceptable fit to the data (CFI= .965; TLI = .958; RMSEA = .029). Further, we created separate cross-longitudinal models of the relationship between SR and SWB (*Table 5*).

**Table 5:** Cross-lagged models

Model	X^2^	df	CFI	RMSEA	TLI
Cross-lagged (T1_SR → T2_SWB)	90.420	83	0.984	0.026	0.976
Cross-lagged (T1_SWB → T2_SR)	106.355	89	0.962	0.039	0.948
Cross-lagged (T2_SWB → T3_SR)	103.781	92	0.973	0.031	0.965
Cross-lagged (T2_SR → T3_SWB)	94.256	92	0.995	0.014	0.993

All models turned out to be significant, with their conformity indices demonstrating high levels of significance. To identify the direction of the cross-longitudinal relationships, we compared the significance and magnitude of the standardized regression coefficients in the models (*Table 6*).

**Table 6:** Auto- and cross-lagged standardized path coefficients from the cross-lagged panel analyses

	SWB Time 2	SWB Time 3	SR Time 2	SR Time 3
SWB Time 1	0.42***		0.21**	
SWB Time 2	1	0.20**		***-0.06***
SR Time 1	0.28**		0.43***	
SR Time 2		0.41***	1	0.82**

Notes. *p < .05; **p < .01; ***p < .001. *Cross-longitudinal coefficients are in bold italics*

Autoregressive coefficients for the SWB indicators were lower than for SR, which testified to a greater variability of the school SWB indicators for adolescents. Evaluation of the cross-longitudinal relationships revealed that when students were moving from grade 4 to grade 5 (Time 1 and Time 2), the relationship between SR and SWB was reciprocal. The cross-longitudinal relationship between SR and SWB during the transition from grade 5 to grade 6 (Time 2 and Time 3) was significant only for SR. Th at is, we can say that a higher level of self-regulation development among students in primary school predicted their well-being in the 5^th^ grade, and then in the 6^th^ grade.

## Discussion

Comparative analysis of the longitudinal data made it possible to establish that there are certain differences in the dynamics of changes in the PWB and SWB levels of adolescents during their transition from primary to basic school. The School-Related Subjective Well-Being was characterized by the most pronounced trajectory of change. It had an increasing tendency in the 5^th^ grade, and then decreased in the 6^th^ grade. Psychological Well-Being was characterized by greater stability and slight growth during the period of study from grades 4 to 6. The growth of well-being (both subjective and psychological) from grade 4 to 5, in our opinion, took place due to the changes in educational conditions toward actualizing students’ positive expectations, expanding their spheres of communication, and encouraging initiative and independence in self-organization of activities. During this period, adolescents could acquire skills of well-being that would help them build positive conditions of school life (Ng et al., 2015).

A number of researchers have stressed that primary school years represent a critical period when the students’ experience provides the foundation for their current and later engagement, achievement, and sense of belonging in school (Suldo et al., 2014; Tian et al., 2016). As we did, these researchers noted that puberty is characterized by decreasing SWB, and that adolescents demonstrate a clear drop in the life satisfaction (Shek & Liu, 2014; Steinmayr et al., 2018; Willroth et al., 2020). Furthermore, after the end of puberty, their sense of well-being increases (Salmela-Aro & Tuominen-Soini, 2010).

However, we can identify somewhat different dynamics for PWB and SWB. There was evidence that PWB is indeed more sustainable over the long term (Joshanloo, 2018). It is definitely PWB that determined the level of school-related SWB (the cause-and-effect relationship mainly goes from PWB to SWB, and not vice versa). In this sense, we have confirmed these conclusions.

The trajectory of conscious SR development in both studies was characterized by smooth growth. An increase of the regulatory indicators took place largely due to age characteristics and the social situation for development. In turn, self-regulation became a resource for personal growth in adolescence (Bronson, 2000; Morosanova, Bondarenko, Fomina & Burmistrova-Savenkova, 2018).

The relationships between PWB and SR which we uncovered demonstrated the heterogeneity and heterochrony of these properties’ development in adolescents. As a whole, the PWB indicators were steadily increasing, and the indicators of conscious SR, rising in the 5th grade, returned to their previous values in the 6th grade. The results of the analysis revealed that high PWB level in the 4th grade predicted a high SR level in the 5th grade.

These results are consistent with the general conclusions of researchers that PWB affects a wide range of factors in the lives of children and adolescents. A high level of personality and events management, high self-esteem, well-built relationships with teachers and peers, and mental balance allow 4^th^-graders to successfully develop their self-regulation. The 5^th^ grade is associated with the transition to new learning conditions. These circumstances challenge the independence of schoolchildren and their ability to regulate their activities.

Cross-longitudinal analysis made it possible to record more significant effects of SR on PWB than of PWB on SR. In other words, the child’s PWB level in grade 5 did not significantly affect his/her SR in grade 6. It can be concluded that the PWB level achieved in the 5th grade at the stage of adaptation to new learning conditions served as the foundation for development of conscious SR. Upon completion of the adaptation process, the conscious SR serves as a significant resource for maintaining the adolescents’ PWB in the basic secondary school. Thus, during the transition from primary to basic secondary school, PWB can be a significant condition for conscious SR development. In turn, SR acts as a resource for the growth of PWB indicators in the future.

The longitudinal relationship between SR and school-related SWB was characterized by somewhat different features. It should be noted that our study was assessing the cognitive component of school SWB, *i.e.*, life satisfaction. This component is considered more stable, and is most frequently included in studies of youths’ perceived quality of life (Suldo et al., 2006). Thus, during the transition from grade 4 to grade 5, the contributions of SR to school-related SWB and *vice versa* are commensurate, *i.e.,* there is a reciprocal relationship between them. When the child moves from grade 5 to grade 6, however, the situation changes dramatically; conscious SR makes a more significant contribution to SWB, while the contribution of SWB to SR is insignificant. This result is especially interesting because life satisfaction decreases during adolescence, which can influence many later life outcomes (Blakemore & Mills, 2014; Orben et al., 2020). Thus, conscious SR acts as a SWB resource throughout the entire schooling period. And its contribution to well-being is especially significant during the transition from 5th to 6th grade.

Accordingly, reliance on internal resources becomes important (Goldbeck, Schmitz, Besier, Herschbach, & Henrich, 2007; Steinmayr et al., 2018). During this period, adolescents demonstrate a decline in academic motivation, which inevitably leads to a decrease in academic performance, which cannot but affect school-related SWB (Martin, & Steinbeck, 2017). The relationship between academic achievement and school-related SWB weakens significantly during adolescence (Yang et al., 2019), while conscious SR still remains a reliable predictor of both academic achievement and SWB in adolescents (Fomina & Morosanova, 2019; Gestsdottir & Lerner, 2008). Thus, conscious self-regulation, being a foundation for success in educational activity, is a necessary resource for maintaining the SWB of adolescents in this difficult age period.

## Conclusion

A three-year longitudinal study demonstrated a stable relationship between conscious self-regulation and both psychological and subjective school-related well-being in adolescence.

Cross-longitudinal analysis then made it possible to establish reciprocal relationships between conscious SR and PWB. It has been shown that during the transition from primary to basic secondary school, PWB can act as a significant mechanism for the development of SR. However, in the future, conscious SR can be considered as a significant resource for maintaining adolescents’ PWB in subsequent years.

The longitudinal relationships between SR and school-related SWB were characterized by slightly different specificity. The general level of the conscious self-regulation of educational activity in adolescents predicted the level of their subjective wellbeing to a greater extent, and, in this sense, acted as an effective tool for maintaining well-being in adolescence.

## Limitations

The present research did not set the task of studying gender differences in the dynamics of SWB, PWB, and SR, although, according to previous research, it is necessary to take gender specificity into account in this context (Orben et al., 2020). In addition, certain individual characteristics of adolescents can also play a significant role in the dynamics of the studied phenomena. The study of these issues will form the basis for our future research.
